# IUC24410-76 Early PSA suppression with adjuvant abiraterone in high-risk prostate cancer: real-world data on the achievement of ultralow PSA levels

**DOI:** 10.1093/oncolo/oyaf248.013

**Published:** 2025-08-29

**Authors:** F Di Costanzo, D Alaimo, R Pearson, J Frew, X Jiang, R Plummer, A Greystoke, C Oing, R Chandler, P Rescigno

**Affiliations:** Translational and Clinical Research Institute, Centre for Cancer, Newcastle University, Newcastle upon Tyne, Italy; Department of Internal Medicine and Medical Therapy, University of Pavia, Italy; Northern Centre for Cancer Care, Freeman Hospital, Newcastle upon Tyne, Italy; Northern Centre for Cancer Care, Freeman Hospital, Newcastle upon Tyne, Italy; Northern Centre for Cancer Care, Freeman Hospital, Newcastle upon Tyne, Italy; Translational and Clinical Research Institute, Centre for Cancer, Newcastle University, Newcastle upon Tyne, Italy; Translational and Clinical Research Institute, Centre for Cancer, Newcastle University, Newcastle upon Tyne, Italy; Translational and Clinical Research Institute, Centre for Cancer, Newcastle University, Newcastle upon Tyne, Italy; Northern Centre for Cancer Care, Freeman Hospital, Newcastle upon Tyne, Italy; Translational and Clinical Research Institute, Centre for Cancer, Newcastle University, Newcastle upon Tyne, Italy

## Abstract

**Background:**

Achieving an ultralow PSA nadir (<0.02 ng/mL) after radical treatment is increasingly recognized as a surrogate for improved long-term outcomes in high-risk non-metastatic prostate cancer. While the STAMPEDE trial demonstrated the efficacy of abiraterone in this setting, real-world evidence remains limited. We report real-world data on the biochemical response to adjuvant abiraterone plus androgen deprivation therapy (ADT), focusing on PSA dynamics and timing of ultralow PSA achievement.

**Methods:**

We retrospectively analyzed 137 patients treated with adjuvant abiraterone/prednisolone plus ADT across UK NHS centers between 2023 and 2025. Evaluated outcomes included the rate and timing of PSA suppression to < 0.02 ng/mL. Kaplan-Meier analysis and multivariable logistic regression were used to evaluate predictors of achieving PSA response and time to nadir, stratified by abiraterone treatment discontinuation status.

**Results:**

A PSA <0.02 ng/mL was reached in 84.7% of patients (116/137), including 87.5% of those who completed therapy and 75.8% of those who discontinued early (*P* = .162). Median time to ultralow PSA was 5 months in the continuation group and 6 months in those who discontinued (HR = 0.61; 95% CI, 0.39-0.96; *P* = .031). Among those who discontinued abiraterone, 5 patients (15.2%) reached the PSA threshold after treatment interruption. Notably, in 90% of cases, PSA <0.02 ng/mL was achieved within 12 months of initiating therapy. No clinical variables were independently associated with achieving PSA <0.02 ng/mL.

**Conclusions:**

In this real-world cohort, the majority of high-risk patients achieved early and profound PSA suppression with adjuvant abiraterone, even when treatment was discontinued prematurely. These findings support the biological efficacy of abiraterone and suggest that early PSA nadir may guide future response-adapted treatment strategies.

**Table:** 

Discontinuation	PSA < 0.02 ng/mL	PSA ≥ 0.02 ng/mL	p-value (Fisher)
No	91	13	0.162
Yes	25	8	

